# Correction: A Trial on the Effects of Magnesium-Zinc-Calcium Vitamin D Co-supplementation on Glycemic Control and Markers of Cardio-metabolic Risk in Women with Polycystic Ovary Syndrome

**DOI:** 10.34172/aim.2022.92

**Published:** 2022-08-01

**Authors:** Mehri Jamilian, Maryam Maktabi, Zatollah Asemi

**Affiliations:** ^1^Endocrinology and Metabolism Research Center, Department of Gynecology and Obstetrics, School of Medicine, Arak University of Medical Sciences, Arak, Iran; ^2^Research Center for Biochemistry and Nutrition in Metabolic Diseases, Kashan University of Medical Sciences, Kashan, I.R. Iran

 This corrects the article: “A trial on the effects of magnesium-zinc-calcium vitamin D co-supplementation on glycemic control and markers of cardio-metabolic risk in women with polycystic ovary syndrome”1 published on “2017;20(10):640-645” in the Archives of Iranian Medicine journal.

 In the original version of this article, participants was wrongly reported 60 in the results, while the correct participants is 30 in each group.

 In addition, in the conclusions section, a citation from our previous paper was omitted. The correct conclusions are another article by Maktabi et al39 with the same clinical trial described identical study timelines, location, sample size, randomization methods showed that co-supplementation had beneficial effects on hormonal profiles, biomarkers of inflammation, and oxidative stress.

 39. Maktabi M, Jamilian M, Asemi Z. Magnesium-Zinc-Calcium-Vitamin D Co-supplementation Improves Hormonal Profiles, Biomarkers of Inflammation and Oxidative Stress in Women with Polycystic Ovary Syndrome: A Randomized, Double-Blind, Placebo-Controlled Trial. Biol Trace Elem Res. 2018 Mar; 182(1):21-28.
